# A Rare Triad of Pulmonary Embolism, Inferior Vena Cava Thrombosis, and Hepatic Cyst Compression Revealing Occult Non-small Cell Lung Cancer

**DOI:** 10.7759/cureus.94296

**Published:** 2025-10-10

**Authors:** Agnieszka Gryguc-Saxanoff, Allison Reichl

**Affiliations:** 1 Hospital Medicine, Ronald Reagan University of California Los Angeles Medical Center, Los Angeles, USA; 2 Internal Medicine, University of California Los Angeles, Los Angeles, USA

**Keywords:** acute pulmonary embolism, compression of ivc, hepatic lesion, inferior vena cava (ivc) thrombosis, malignancy-associated hypercoagulability, metastatic non-small cell lung cancer

## Abstract

We present the case of a 74-year-old woman who was admitted with acute shortness of breath and ultimately diagnosed with bilateral pulmonary emboli, inferior vena cava thrombosis, and a rapidly enlarging hepatic cyst. The cyst was compressing the inferior vena cava and contributing to thrombus formation. Her hospital course was further complicated by a diagnosis of metastatic non-small cell lung cancer harboring an epidermal growth factor receptor exon 19 deletion. This particular clinical scenario highlights the importance of maintaining a broad differential diagnosis when assessing pulmonary embolism, particularly in the absence of clear provoking factors. It illustrates how vascular obstruction, mass effect from benign lesions, and malignancy-associated hypercoagulability can converge to create a complex clinical picture.

## Introduction

Pulmonary embolism is a common cause of morbidity and mortality among hospitalized patients and often arises from deep vein thrombosis through mechanisms described by Virchow’s triad: impaired venous return (stasis), a hypercoagulable state, and vascular injury. In some instances, pulmonary embolism may be the first clinical manifestation of an underlying malignancy. Cancer is a well-recognized driver of a prothrombotic state, often serving as the sole cause of venous thrombosis and embolism through tumor-related expression of procoagulant factors and systemic inflammation [1-3]. Thrombosis involving the inferior vena cava is less common than thrombosis in the lower extremity veins. However, it can still carry profound clinical implications, particularly when mechanical compression from adjacent structures is also present. Hepatic cysts are frequently incidental findings and usually asymptomatic. Still, when they enlarge, they may compress nearby structures such as the inferior vena cava or bile ducts, resulting in venous stasis, thrombosis, or biliary obstruction [4,5]. Lastly, such a symptom profile becomes even more complex when these anatomical factors coexist with an occult malignancy, which not only promotes a hypercoagulable state but also complicates timely diagnosis and management.

## Case presentation

**Figure 1:**
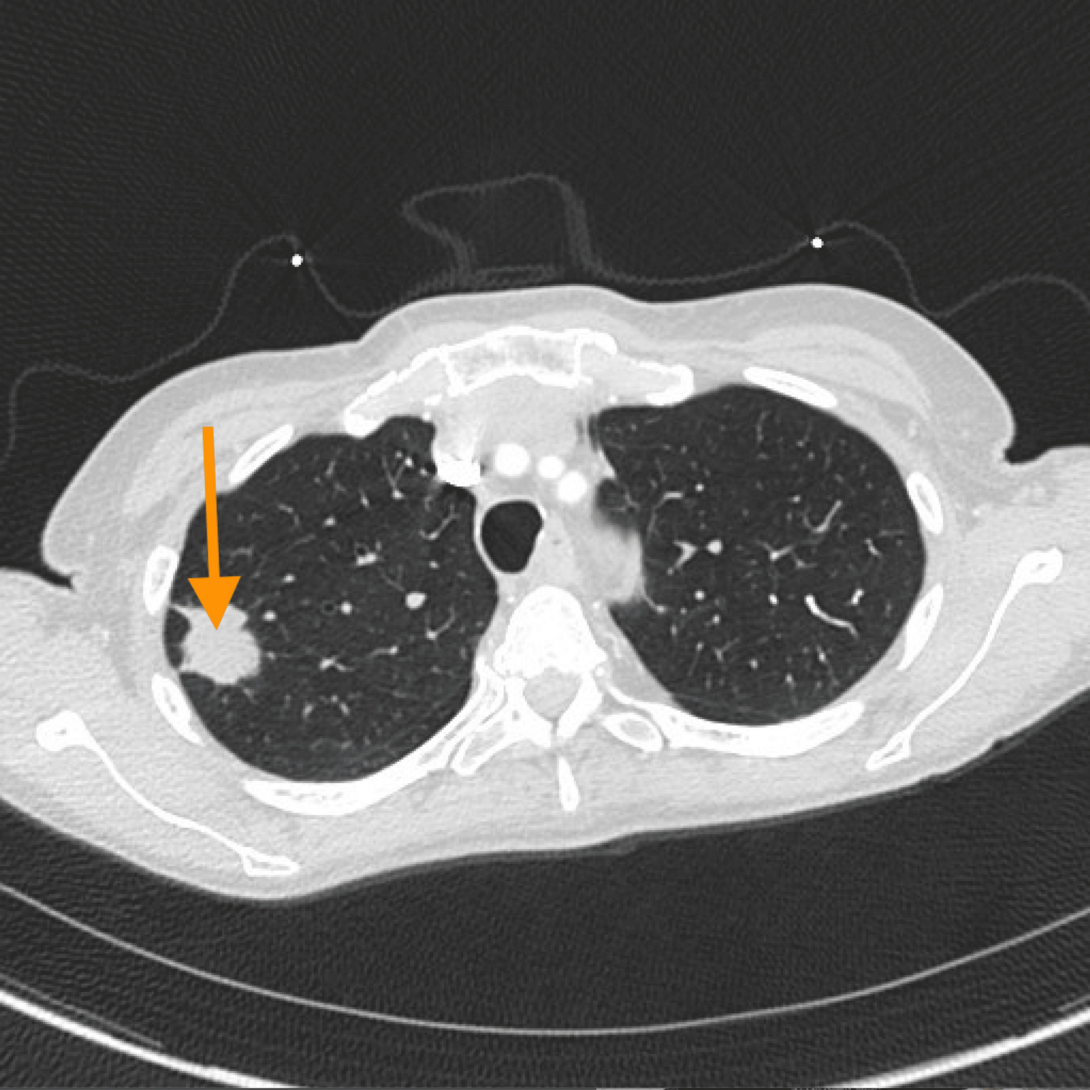
Lung nodule in the right upper lobe

A 74-year-old woman with a medical history of reactive airway disease presented to the emergency department with sudden-onset shortness of breath. Upon history taking, it was noted that she was a lifelong nonsmoker, denied alcohol or illicit drug use, and had no known personal or family history of cancer or thromboembolic disease. She reported several months of unintentional weight loss but denied chest pain, palpitations, fever, or recent illness. Several days prior, a chest computed tomography (CT) scan performed for an unrelated concern had identified a right upper lobe pulmonary mass (size >3 cm). No evidence of metastatic disease was seen on this initial imaging. At that time, a biopsy had been scheduled (Figure 1).

**Figure 2:**
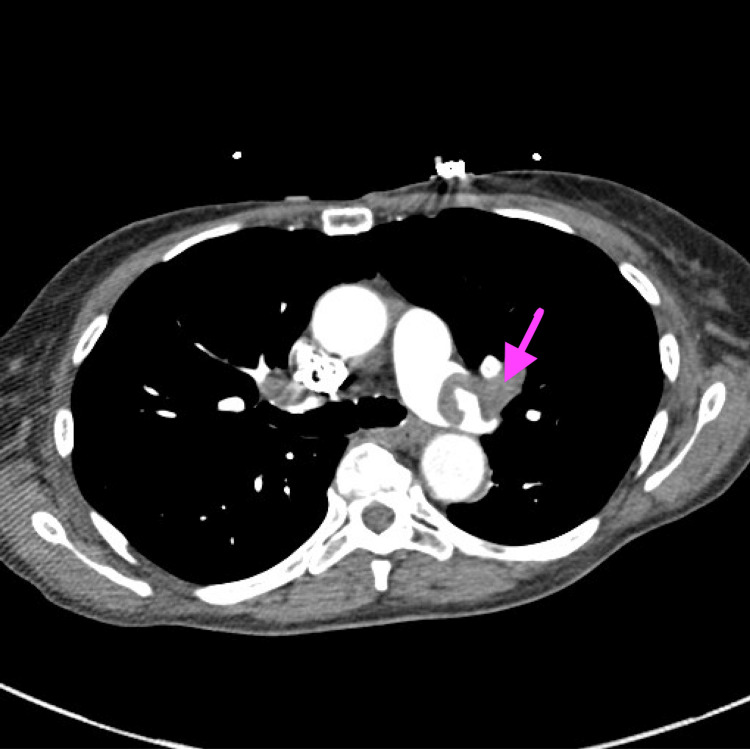
An arrow with pulmonary embolism in the left main pulmonary artery

On arrival, her heart rate was 110 beats per minute, blood pressure was 129/86 mmHg, and she required three liters of supplemental oxygen to maintain adequate oxygen saturation. The patient was not on any form of anticoagulation before her evaluation. CT angiography (CTA) of the chest was significant for large bilateral pulmonary emboli, and transthoracic echocardiography demonstrated signs of right heart strain (Figure 2).

Laboratory testing showed elevated high-sensitivity troponin and B-type natriuretic peptide levels. Within 48 hours, her hemoglobin concentration declined significantly, prompting further investigation (Table 1).

**Table 1:** Selected laboratory findings during hospitalization BNP: B-type natriuretic peptide

Test	Day 1	Day 3	Reference Range	Interpretation
Hemoglobin (g/dL)	11.2	8.0	12.0–15.5	Significant drop
Troponin (ng/L)	1122	–	<14	Elevated
BNP (pg/mL)	537	–	<100	Elevated

**Figure 3:**
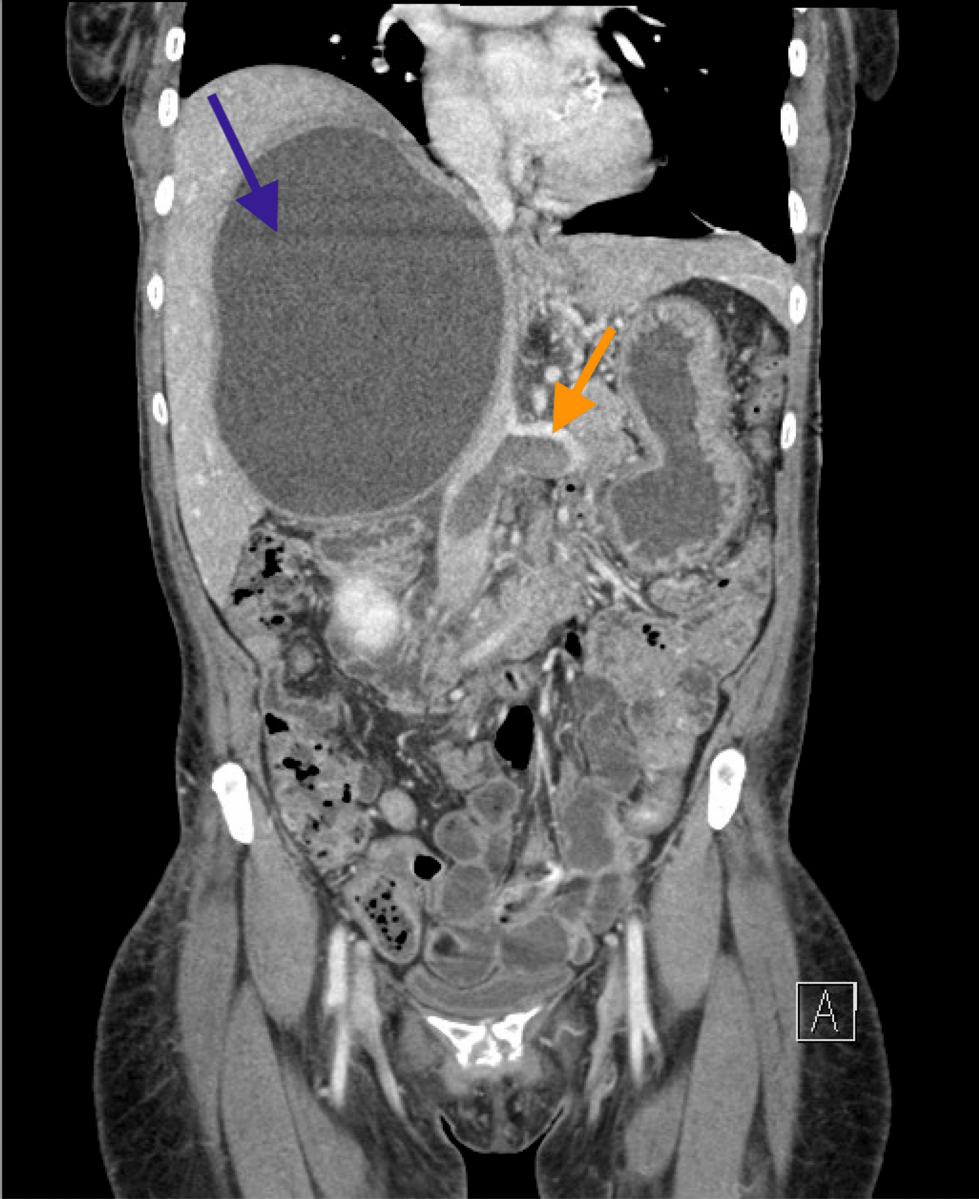
A CT of the abdomen and pelvis (venous phase) obtained on hospital day 3 The blue arrow shows a 16 cm hepatic cyst causing a mass effect on the inferior vena cava. The orange arrow indicates the extensive thrombus located below the cyst, as visualized in the inferior vena cava and left renal vein.

An abdominal CTA ruled out intra-abdominal hemorrhage but revealed an occlusive thrombus in the inferior vena cava, extending into the renal and iliac veins. A 16-centimeter hepatic cyst was also noted, exerting a mass effect on the inferior vena cava and suspected to be contributing to thrombosis through compression (Figure 3).

To reduce the risk of thrombus migration, she underwent catheter-directed thrombolysis, followed by inferior vena cava thrombectomy and aspiration of the hepatic cyst. The aspirated cyst fluid tested negative for malignant cells and infection, and serologic testing for echinococcosis was also negative. She was discharged on therapeutic anticoagulation with enoxaparin.

At her outpatient follow-up visit, a biopsy of the pulmonary mass confirmed non-small cell lung cancer. Positron emission tomography (PET) combined with CT demonstrated increased fluorodeoxyglucose (FDG) uptake in segment 8 of the liver and the hilar lymph nodes. A subsequent liver biopsy confirmed metastatic non-small cell lung cancer, while the hepatic cyst remained benign. Molecular testing revealed an epidermal growth factor receptor exon 19 deletion, and the patient was started on osimertinib 80 mg daily approximately three months after her initial hospitalization.

## Discussion

Hepatic cysts are usually benign and asymptomatic; however, when they enlarge, as in this case, measuring 16 cm, or are situated near critical structures, they can lead to clinically significant problems. In rare circumstances, they compress adjacent vessels such as the inferior vena cava, resulting in impaired venous return, venous stasis, and thromboembolism. Previous reports describe cases where giant hepatic cysts produced inferior vena cava syndrome, with symptoms relieved following cyst drainage and surgical intervention [6-8]. In this patient, there was no evidence of metastatic disease on the initial imaging. The thrombus likely formed through a combination of mechanical compression from the hepatic cyst and a hypercoagulable state driven by an underlying, undiagnosed non-small cell lung cancer. Malignancy is a well-established cause of venous thromboembolism and may serve as the sole driver of thrombosis through tumor expression of procoagulant factors, systemic inflammation, and vascular injury [1-3]. Pulmonary embolism may be the first clinical clue to an occult cancer, and up to 10% of patients with unprovoked pulmonary embolism are ultimately diagnosed with malignancy within the year [9]. Moreover, venous thromboembolism in the setting of cancer is associated with worse outcomes overall [10]. The patient underwent a multidisciplinary treatment strategy, including catheter-directed thrombolysis, inferior vena cava thrombectomy, and aspiration of the hepatic cyst. These interventions not only reduced clot burden and prevented embolic complications but also relieved local compression. In more complex cases, surgical approaches such as laparoscopic fenestration or resection may be necessary, particularly when cyst size or recurrence limits the efficacy of aspiration alone [4,5]. Elaboration of the surgical aspect is relevant here, as it underscores that mechanical obstruction must sometimes be addressed in tandem with systemic anticoagulation and oncologic therapy. Taken together, this case illustrates a rare but essential presentation of malignancy: venous thromboembolism arising from the convergence of inferior vena cava compression, cyst-related mass effect, and cancer-associated hypercoagulability. Vascular complications of inferior vena cava compression, including thromboembolism, venous stasis, and impaired blood flow, should be recognized promptly, as they may guide the order and choice of intervention. Recognition of this multifactorial process required close coordination between interventional radiology, hepatobiliary surgery, pulmonology, and oncology. A multidisciplinary approach was crucial for stabilizing and initiating targeted oncologic therapy promptly.

## Conclusions

Clinicians should maintain a high index of suspicion for occult malignancy when encountering adult patients (older adults in particular) presenting with unprovoked pulmonary embolism. Although hepatic cysts are generally benign, they can become clinically significant when they are large enough to compress vascular structures, such as the inferior vena cava. When a cyst contributes to thrombosis, thrombolytic therapy or thrombectomy should precede drainage to reduce the risk of embolic complications. Effective management of these complex presentations requires a multidisciplinary approach involving multiple specialties.

## References

[1] Timp JF, Braekkan SK, Versteeg HH, Cannegieter SC (2013). Epidemiology of cancer-associated venous thrombosis. Blood.

[2] Falanga A, Marchetti M, Vignoli A (2013). Coagulation and cancer: biological and clinical aspects. J Thromb Haemost.

[3] Blom JW, Doggen CJ, Osanto S, Rosendaal FR (2005). Malignancies, prothrombotic mutations, and the risk of venous thrombosis. JAMA.

[4] European Association for the Study of the Liver (2022). EASL clinical practice guidelines on the management of cystic liver diseases. J Hepatol.

[5] Chan YC, Morales JP, Reidy JF, Taylor PR (2008). Management of spontaneous and iatrogenic retroperitoneal haemorrhage: conservative management, endovascular intervention or open surgery?. Int J Clin Pract.

[6] Nakabayashi K, Murakami M, Hata S (2021). Giant hepatic cyst: a possible cause of inferior vena cava syndrome. Intern Med.

[7] Ko MK, Kim T, Lee WH, Park SH, Choi JH, Shin M, Heo NY (2018). Deep vein thrombosis due to compression of a huge hepatic cyst was successfully treated by an inferior vena cava filter and cyst drainage (Article in Korean). Korean J Gastroenterol.

[8] Mammone S, Cozzolino A, Amoruso DC (2023). Giant hepatic cyst determining secondary Budd-Chiari syndrome: cruciality of ultrasound as a diagnostic and therapeutic tool. Ultrasound J.

[9] Monreal M, Lafoz E, Casals A (1991). Occult cancer in patients with deep venous thrombosis: a systematic approach. Cancer.

[10] Sørensen HT, Mellemkjaer L, Olsen JH, Baron JA (2000). Prognosis of cancers associated with venous thromboembolism. N Engl J Med.

